# Commentary: On the levels of patient selection in registry-based randomized controlled trials

**DOI:** 10.1186/s13063-019-3214-x

**Published:** 2019-02-04

**Authors:** Florian Lasch, Kristina Weber, Armin Koch

**Affiliations:** 1 Department of Biostatistics, Hannover Medical School, Carl-Neuberg Strasse 1, 30165 Hannover, Germany; 2 F. Hoffmann-La Roche AG, MDBD, Bldg 663, Hochstrasse, CH-4070 Basel, Switzerland

**Keywords:** Registry, Registry-based RCT, Patient selection, Pragmatic trials, Generalizability, External validity

## Abstract

Registry-based randomized controlled trials (RCTs) are presumed to include a less-selected patient population and thus to have enhanced generalizability compared to conventional RCTs. However, this view disregards the levels of patient selection in registry-based RCTs: the registry selection level and the trial selection level. At both levels, systematic selection can occur and generalizability can be diminished. Nevertheless, using a registry as a basis for recruitment, randomization, and data collection results in an advantage: the trial selection takes place within the registry framework, where baseline characteristics of non-enrolled patients are automatically documented as well. By comparing the baseline variables of the enrolled and non-enrolled patients, the trial selection can always be investigated, which gives a sound basis for discussing the generalizability to the registry population.

## Background

Hypothesized to be “the next disruptive technology in clinical research” [1] and “a new clinical trial paradigm” [2], registry-based randomized controlled trials (rRCTs) are proposed as an answer to important limitations associated with conventional randomized controlled trials (RCTs). Building on registries as a platform for patient recruitment, randomization, and data collection, rRCTs are hoped to lead to reduced costs, rapid consecutive enrolment, and completeness of patient follow-up [3]. Another presumed advantage is enhanced generalizability compared with RCTs by embedding trial recruitment into the clinical routine and including less-selected patient populations [2–5].

On the contrary, Laurer and D’Agostino ask the fundamental questions, whether representativeness can be assured, given that even within a registry there may be a systematic difference between patients who are randomized and those who are not. This ambiguity indicates that the situation is more complicated and calls for a closer look at the alleged generalizability of rRCTs.

In this commentary, we concentrate on different levels of patient selection in rRCTs to achieve a better understanding of the possibilities, limitations, and the generalizability of rRCTs results. Other relevant aspects of the rRCT design such as the choice or endpoints, data quality, or practical implementation are not covered in this article.

## Patient selection in rRCTs

### Levels of patient selection

While internal validity in RCTs is strong, the lack of external validity or a profound assessment of the generalizability is the most frequent critic of RCTs [2, 6–8]. Rothwell identified the patient selection (both, described and beyond what is described by criteria for inclusion and exclusion [9]) as one of the main determinants of the generalizability of trials findings [8]. Whenever trial results are to be generalized to (i.e. applied in) a larger target patient population, erroneous conclusions may occur if the trial population and the target population are structurally different. Since patient selection most often includes structural differences (i.e. regarding baseline characteristics, co-medication, or standard diagnostic measures), the generalizability crucially depends on the (absence of) systematic selection processes. Consequently, the assessment of whether the trial population truly reflects the targeted patient population is an essential step in drug licensing. In instances, subgroups of the target population (e.g. the elderly [10]) were excluded from the approval for the respective treatment if the information from the trial on this subgroup was felt to be too limited and generalizability of the overall trials results to this subgroup could not be assumed.

Problems in the assessment of the external validity of RCTs are hoped to be solved or at least diminished in rRCTs by recruiting patients from an existing patient registry [2–5, 11, 12]. Here, patient registries are defined corresponding with Gliklich and Dreyer as “an organized system that uses observational study methods to collect uniform data (clinical and other) to evaluate specified outcomes for a population defined by a particular disease, condition, or exposure and that serves one or more predetermined scientific, clinical, or policy purposes” [6]. According to our interpretation, this definition includes the requirement that the registry population is complete or representative for the specified population (the registry target population) and respective methods for verification are in place. It is important to note that the existence of a registry protocol (e.g. [13]) is essential for the assessment of the registry properties. Consequently, as a distinction from regular observational studies, in the following, a registry is always assumed to be complete or representative of its target population.

**Fig. 1:**
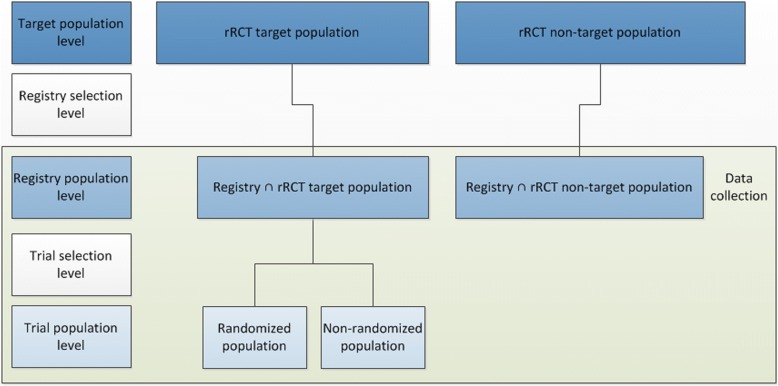
Levels of patient selection in rRCTs

Figure 1 illustrates the general selection levels in rRCTs, which essentially affect the generalizability of rRCT results. The target population of the rRCT (rRCT target population) is defined as the population to which the study findings are meant to apply by the investigators [6]. The registry (e.g. a disease registry) supposed to host the rRCT is typically not developed for the trial and likely has a different target population (registry target population). Here, the registry protocols play an essential role for the evaluation of the aims and scope of the registry and the respective selection mechanism. On the one hand, the registry might have a broader scope than the RCT and comprise patients who are not in the RCT target population. For example, a heart failure registry might include patients with New York Heart Association (NYHA) functional classifications II, III, and IV, but the RCT based on this registry only includes patients with NYHA functional classifications III and IV, excluding patients with the mildest symptoms. On the other hand, some patients of interest for the treatment comparison of the RCT might not be included in the registry. For example, only adults could be included in the registry, although the treatment would also be an option for children and adolescents. Another problem arises if the registry does not cover all geographic regions and healthcare systems in which trial results are meant to be applied [8, 14–16]. Additionally, the selection of participating institutions is an important factor: if only large-scale academic hospitals or hospitals with a minimum caseload per year participate in the registry, the structural exclusion of a subpopulation of patients from the registry might be induced. Thus, generally, the rRCT target population included into the registry is a strict subset of the rRCT target population, which can be defined as registry selection. Additionally, the trial population is recruited from the intersection of the registry population and the rRCT target population. If these populations are not congruent (since, for example, some patients from the registry are unwilling to participate in the trial, do not meet the criteria for inclusion and exclusion, or are lost to follow-up for systematic reasons), trial selection processes are present. Ultimately, there are two levels of patient selection when conducting an rRCT:

i) the registry selection: the registry population is selected from the rRCT target population;
ii) the trial selection: the trial population is selected from the registry population.

### Analysis of the representativeness

Li et al. [3] and James et al. [2] assume that a less-selected patient population and thus enhanced generalizability is automatically assured in rRCTs. However, the analysis of the levels of patient selection reveals a more complex picture. Both levels of selection influence the generalizability of the rRCT to the registry target population. While the trial selection level applies for all rRCTs, the registry selection level depends on the design. Given the scientific question of the rRCT and a respective target population, the registry can be defined as all-inclusive from the trial perspective if the complete rRCT target population is included and followed up [2]. For example, the Swedish angiography and angioplasty registry (SCAAR) [17] or the Nordic prescription databases [18] are all-inclusive regarding inferences about the five Nordic countries. However, the SCAAR cannot be considered all-inclusive regarding all of Europe. If, and only if, an all-inclusive registry is used as a platform for the rRCT, the registry selection does not apply. This aspect crucially affects the generalizability of an rRCTs: if the registry target population does not include the complete rRCT target population, the original research question cannot be answered without extrapolation. However, even in the best-case scenario that an appropriate all-inclusive registry is used, the trial selection level applies. Consequently, in all scenarios, the generalizability of the trial results to the registry target population is not automatically given and has to be investigated.

For analyzing the patient selection of conventional RCTs, it is necessary to evaluate information on the screened patients and the target population [19, 20]. The same is needed to investigate the registry selection level: at least the definition of the registry target population and selection mechanisms based on the registry protocol and the criteria for inclusion and exclusion of the trial have to be compared. For assessing the trial selection level, however, no external information is needed. As shown in Fig. 1, the trial selection level takes place within the registry framework, where baseline characteristics of non-enrolled patients are documented by default. By comparing the baseline variables of the enrolled and non-enrolled patients, the trial selection can be described, and substantial differences between the trial population and the complement in the registry can be investigated. Thereby, the difficulty in determining the number and characteristics of eligible non-randomized patients, which was identified by Rothwell as a major problem in the assessment of the representativeness of conventional RCTs [8], is solved.

### Adequate comparisons

It is important to note that comparing enrolled and non-enrolled patients regarding any outcome variable, which is influenced by treatment decisions (randomized versus non-randomized), is potentially biased. The first differentiating factor between the trial patients and the registry-only patients is the fact that only the former have been included in the trial and likely there is a clinical rationale. The second differentiating factor is the treatment allocation mechanism applied: in the enrolled group, treatment is allocated randomly, while in the non-enrolled group the treating physician decides about the treatment. Thus, enrolled and non-enrolled patients differ structurally regarding at least two factors and a difference between these groups observed after treatment allocation cannot be causally attributed to only one of the factors. Consequently, no variable measured after the treatment decision (or treatment allocation in the trial, respectively) should be used for the assessment of patient selection and generalizability. Only a comparison regarding baseline characteristics allows an unbiased assessment of structural differences between the populations to evaluate the patient selection of the rRCT from the utilized registry.

## Example: TASTE trial

### TASTE trial: background and design

The rRCT design was applied in the Thrombus Aspiration during ST-segment Elevation myocardial infarction (TASTE) trial. This trial is based on the SCAAR platform, which holds data on consecutive patients from all 29 Swedish, one Icelandic, and one Danish coronary intervention centers [21]. TASTE was conducted to evaluate the clinical effect of routine intracoronary thrombus aspiration before primary percutaneous coronary intervention (PCI) compared to PCI alone in patients with ST-segment elevation myocardial infarction (STEMI). For this multicenter, prospective, open-label RCT 7244 patients were enrolled from SCAAR, while 4712 eligible patients registered in SCAAR were not enrolled [22, 23].

### Comparison of the randomized and non-randomized populations

As explained in section 'Patient selection in rRCTs', the trial selection within the registry can be investigated in an rRCT. We assess trial selection level by comparing the non-randomized patients from SCAAR to the trial population based on the baseline characteristics provided by Lagerqvist et al. [22]. Within the trial population and the complement in the registry, patients are pooled regarding the received treatment (PCI + thrombus aspiration or PCI alone). Here, the focus is on the effect estimates and confidence intervals rather than on the *p* values, because the *p* values can indicate structural differences for clinical non-relevant effects due to the large sample sizes of both populations. The population comparison regarding baseline characteristics (see subsection 'Adequate comparisons') shows that the randomized and the non-randomized population differ regarding important prognostic factors (see Table 1). It is not only the proportion of male patients that is higher in the randomized group (74.9% vs 67.9%; risk difference [RD] – 6.9%; 95% confidence interval [CI] [− 8.6%, − 5.3%]). More importantly, the proportions of patients with Killip class ≥ 2 (6.6% vs 16.9%; RD 10.2%; 95% CI [9.0%, 11.4%]) and the proportion of patients with previous myocardial infarction (11.62% vs 17.7%; RD 6.1%; 95% CI [4.8%, 7.4%]) are more extensive in the non-randomized patients by statistically significant differences. These differences reveal that the non-randomized patients have been different from the randomized patients from the beginning and trial selection took place. As mentioned by Fröbert et al., this could be because 37.6% of the non-enrolled patients were not able to provide oral informed consent mainly owing to severe medical conditions [22, 23]. This observation in itself strongly hints at substantial differences between both populations.

**Table 1:** Comparison of baseline characteristics between the TASTE population and the non-randomized SCAAR population

Endpoint	Randomized patients^a^ (*n* = 7244)	Non-randomized patients^a^ (*n* = 4712)	Comparison of randomized and non-randomized patients^b^	*p* value^c^
Age (years) (mean ± SD)	66.2 (±11.6)	68.8 (±12.8)	MD = 2.6 [2.1, 3.0]	< 0.001
Male sex (n (%))	5424/7244 (74.9)	3201/4712 (67.9)	RD = −6.9% [−8.6, − 5.3]	< 0.001
Diabetes mellitus (n (%))	901/7244 (12.4)	799/4712 (17.0)	RD = 4.5% [3.2, 5.8]	< 0.001
Current smoking (n (%))	2256/7244 (31.1)	1198/4712 (25.4)	RD = − 5.7% [− 7.4, − 4.1]	< 0.001
Previous myocardial infarction (n (%))	842/7244 (11.6)	836/4712 (17.7)	RD = 6.1% [4.8, 7.4]	< 0.001
Previous PCI (n (%))	699/7244 (9.7)	572/4712 (12.1)	RD = 2.5% [1.3, 3.6]	< 0.001
Previous CABG (n (%))	144/7244 (2.0)	233/4712 (4.9)	RD = 2.3%, [2.3, 3.7]	< 0.001
Fibrinolysis before PCI (n (%))	137/7244 (1.9)	116/4712 (2.5)	RD = 0.6%, [0.0, 1.1]	0.039
Killip class ≥ 2 (n (%))	481/7244 (6.6)	794/4712 (16.9)	RD = 10.2%, [0.9, 11.4]	< 0.001
Radial-artery approach (n (%))	4809/7244 (66.4)	2588/4712 (54.9)	RD = − 11.5% [− 13.3,-9.7]	< 0.001
Type of disease (n (%))				< 0.001
One-vessel disease	3886/7244 (53.5)	2192/4712 (46.5)	RD = − 7.1% [− 9.0, − 5.3]	
Two-vessel disease	2082/7244 (28.7)	1258/4712 (26.7)	RD = − 2.0% [− 3.7, − 0.4]	
Three-vessel disease	1056/7244 (14.6)	874/4712 (18.6)	RD = 4.0% [2.6, 5.3]	
Left main coronary artery disease	203/7244 (2.8)	366/4712 (7.8)	RD = 5.0% [4.1, 5.8]	
Data not available	17/7244 (0.2)	22/4712 (0.5)	RD = 0.2% [0.0, 0.4]	

^a^Continuous variables are pooled by calculating the sample size weighted means and pooled standard deviations

^b^For continuous endpoints mean difference (MD; non-randomized - randomized) is calculated with the 95% confidence interval. For binary endpoints, risk difference (RD; non-randomized - randomized) is given with the 95% confidence interval

^c^For continuous endpoints, the *p* value is calculated using a two-sample t-test assuming equal variances; for binary and categorical endpoints the *p* value is calculated for the null hypothesis RD = 0 or equal distributions, respectively, using a chi-squared test without continuity correction

### Lessons learned from the TASTE trial

The investigators’ evaluation of the generalizability of the findings in the randomized trial to the overall registry population was inconsistent. In an early publication [23], the investigators recognized that “a comparison of the clinical characteristics and outcomes between the patients who underwent randomization and those who did not indicates that the two cohorts differed significantly in a number of respects […].” However, this analysis primarily refers to a difference in 30-day all-cause mortality, which was used as the primary efficacy endpoint in the trial and, therefore, is influenced by the treatment decision. As shown in subsection 'Adequate comparisons', this comparison is biased and invalid for the analysis of trial selection. Later, Lagerqvist et al. [22] stated that “on a national basis, the TASTE trial enrolled a very high proportion of all the patients with STEMI for whom PCI was planned and who were eligible to provide oral informed consent.” They concluded that “the trial is therefore truly representative of the overall population of patients in our region with STEMI who undergo PCI.” This is in stark contrast to the detection of substantial patient-selection in subsection 'Comparison of the randomized and non-randomized populations', which indicates that the results from the TASTE trial cannot automatically be generalized to the registry. This systematic patient selection is in line with the registry-based SORT-OUT (Scandinavian Organization for Randomized Trials With Clinical Outcome) trials II–VI, where the randomized and non-randomized patients differ regarding important prognostic factors [24–30].

## Conclusions

RRCTs are hoped to be less selective and thus to have enhanced generalizability compared to conventional RCTs not using registries for recruitment and data collection. The investigation of this alleged advantage through the analysis of selection mechanisms in rRCTs shows that there are two levels of patient selection. Both levels, the registry selection and the trial selection, impact the generalizability. Therefore, rRCTs are neither automatically less selective nor have a higher external validity per se. However, by using a registry as a platform and documenting baseline variables of non-enrolled patients, the rRCT design provides a sound basis for researchers to explicitly investigate the trial selection and thus potential limitations of the generalizability of trial findings to the registry population. This addresses the plea by Rothwell to increase considerations of external validity in trial design and reporting [8]. Such investigations were exemplified based on the TASTE trial, where trial selection diminishes the generalizability to the registry population. For discussing the generalizability to the overall target population, the registry selection level is critical in the design of an rRCT. Only if the complete target population of the rRCT is included in an all-inclusive registry, is it possible to investigate the overall generalizability. In rRCTs based on all-inclusive registries, by default only the trial selection level applies and thus the complete patient selection mechanism can be investigated by analyzing the trial selection. If the registry does not completely cover the rRCT target population, only the trial selection level can be investigated. In this scenario, the generalizability to the overall target population has to be discussed based on external information, as in conventional RCTs, and the original research question cannot be answered without extrapolation. In any case, comparisons of the enrolled and non-enrolled populations should be based on baseline variables, because differences in post-treatment characteristics cannot be attributed definitely to the trial selection.

To conclude, the general advantage of rRCTs in comparison to conventional RCTs is not a less-selected patient population, which is often an illusion, but rather a more thorough ground to investigate the trial selection. The analysis of the trial selection is possible for every rRCT and should be conducted and reported by default to assess the generalizability to the registry population.

## Abbreviations

CI: Confidence interval
NYHA: New York Heart Association
PCI: Percutaneous coronary intervention
RCT: Randomized controlled trial
RD: Risk difference
rRCT: Registry-based randomized controlled trial
SCAAR: Swedish angiography and angioplasty registry
STEMI: ST-segment elevation myocardial infarction
TASTE: Thrombus Aspiration during ST-segment Elevation myocardial infarction trial
